# Bibliometric Analysis and Visualization of Scientific Literature on Heart Disease Classification Using a Logistic Regression Model

**DOI:** 10.7759/cureus.63337

**Published:** 2024-06-27

**Authors:** Neena Suresh, Binu Thomas, Jeena Joseph

**Affiliations:** 1 Department of Computer Sciences, Mahatma Gandhi University, Kottayam, Kottayam, IND; 2 Department of Computer Applications, Marian College Kuttikkanam (Autonomous), Kuttikkanam, IND

**Keywords:** biblioshiny, vosviewer, machine learning, cardiovascular diseases, logistic regression, bibliometric analysis

## Abstract

With the advancement in artificial intelligence, the use of machine learning algorithms for clinical prediction has increased tremendously. Logistic regression is one of the powerful machine learning algorithms that can be used to predict the probability of a variable. Logistic regression is very popular among medical researchers owing to its simplicity, interpretability, and solid statistical foundation. This study aims to investigate the research productivity of heart disease classification using a logistic regression model to analyze the current patterns and potential future trends through bibliometric analysis. Additionally, it aims to highlight the impact and quality of research in the area, identify prominent research groups, the countries actively contributing to the field, which will help the researchers and healthcare professionals to pinpoint research gaps, influential authors, and make informed decisions and invest resources accordingly. The data is collected from a database of Scopus spanning from 2019 to 2023. We have used two bibliometric software, Biblioshiny (Aria and Cuccurullo, 2017) and VOSviewer (Centre for Science and Technology Studies (CWTS), Leiden University, the Netherlands), to analyze the bibliographic data regarding the citation count, contribution of authors, publication count, the contribution of institutions, etc. There are 2331 documents under study which were fed into both software to analyze the data. With 700 documents, China topped the list of most productive countries indicating the vast contribution of the country followed by India and the United States. Contributions of the Harvard Medical School, Boston, MA, United States are found to be the greatest with six papers. The most productive author is Wang Y with 73 documents. Analysis of trending topics reveals that the field progressing towards using support vector machines (SVM), k-nearest neighbours (KNN), and naïve Bayes algorithms.

The article has only considered data from Scopus excluding literature indexed in other databases which limits the potential coverage of the data. Also, the work focuses on recent developments excluding older literature from 2019 which could be a limitation. Furthermore, since the study is a bibliometric analysis targeting the use of logistic regression for heart disease prediction, powerful techniques such as SVM, decision trees, random forests, neural networks and deep learning have not been included, which could be another limitation.

## Introduction and background

A healthy functioning heart is central to the existence of human life. Every cell in our body needs oxygen to produce energy and perform functions. The heart is the primary organ that circulates oxygenated blood to all parts of the body [1]. The anatomical structure of the heart consists of four chambers: there are two atria and two ventricles. The heart works like a pump in our body. The deoxygenated blood from the body arrives at the right atrium. From there, the right atrium squeezes the blood to the right ventricle which pumps it to the lungs, where it becomes oxygenated. The left atrium receives the oxygenated blood from the lungs and gives it to the left ventricle, where the oxygenated blood is pumped to the entire body. Heart disease occurs when plaque is formed in the arteries and blood vessels [2]. Plaque is a waxy material produced by cholesterol, fat molecules, and minerals. High blood pressure, cigarette smoking, or high cholesterol or triglycerides harm an artery's inner lining [3].

Globally, an estimated 523 million people suffer from some type of cardiovascular disease and approximately 19 million succumb to death [4]. Early detection of heart disease can be very crucial for the patient as significant time and a series of tests will be needed for diagnosis [5]. Machine learning (ML) can greatly help in this process by analyzing large amounts of medical data to identify patterns and predict the risks involved. A lot of ML models are used for the clinical prediction of diseases. Logistic regression is a supervised algorithm used for binary classification tasks which predicts the probability of whether a particular instance belongs to a class or not. The heart of the logistic regression model is the sigmoid function. The sigmoid function is defined as:

$\sigma(z)=1/(1+e^{-z})$

where z is a linear combination of the input features and coefficients modelled. The logistic regression model is trained using methods such as maximum likelihood estimation which works by iteratively adjusting the coefficients that minimize the difference between predicted probabilities and original values [1]. Logistic regression is one of the heavily used ML models for disease predictions. Hospitals collect extensive data regarding patients’ vital signs and health metrics which can be used to train artificial intelligence models using logistic regression and predict the probability of having a heart disease. For example, upon arrival of a new patient, during the admission time itself, the model will enable you to identify the risk factor associated with the patient and make appropriate decisions. For example, a patient under high risk could immediately undergo further diagnostic tests or receive proper care [6].

In a study published in 2019, Christodoulou et al. found that there is insufficient evidence to support the claim that clinical prediction models based on ML lead to better area under the curves (AUCs) than those based on logistic regression and concluded that there is no performance benefit of ML over logistic regression for clinical prediction models [7]. In the study, Christodoulou et al. compared prediction models based on logistic regression and ML algorithms for binary outcomes and used AUC as an evaluation metric. Out of 927 articles reviewed, only 71 met the criteria for inclusion which were predominantly from the oncology and cardiovascular medicine fields. They conducted literature research from 2016 to 2017 and emphasized the lack of quality reporting practices and methodology with respect to validation procedures. They have also pointed out that ML models often focus on assessing performance based on discrimination rather than calibration. The results showed that there is no advantage of using ML over logistic regression in clinical domains.

Bibliometric analysis is a quantitative research method used to analyze the publication patterns and characteristics of a particular field, discipline, or research area [8]. It is a systematic method that uses mathematical and statistical tools to analyze bibliographic data to gain insights regarding the research trends, ranging from the contribution of authors to patterns of collaboration among countries, thereby throwing light on interesting and hidden patterns underneath. For example, bibliometric analysis of publications related to artificial intelligence will reveal the rapid growth in the last decade and key fields in this area. Likewise, publications from environmental science will reveal an increasing number of publications regarding climate change and the immediate need for global attention and development of policies on a global level. Bibliometrics has given us a tool that can easily be scaled from the micro (institute) to the macro (world) level [9].

Although logistic regression is widely used in the prediction of heart disease, this is the first bibliometric analysis work that has been carried out in the field. This study intends to shed light on common trends and patterns to identify themes and the direction in which the research area is currently moving. By identifying research gaps and unexplored areas, this study aims to inspire scholars to explore new methods, ultimately assisting patients and healthcare professionals in providing better clinical interventions.

This paper analyzed many key areas including annual scientific production and average citations per year to track the growth in the field along with the impact and relevance of the works published. Three field plot was used to visualize the relationship between authors, keywords and sources using Sankey diagrams. The most relevant sources were tracked to find the prominent journals in the field, enabling the scholars to choose appropriate journals for submission. To discover the geographical distribution and involvement of countries and relevant institutions, the publication output of countries and the contribution of organizations were analyzed. To identify imminent scholars in the field, the most productive authors were checked and to find the most impactful papers in the field, most cited articles were analyzed. Analysis of trending topics and thematic maps showed the emerging trends and patterns in the field. Co-occurrence analysis of index keywords and all keywords provided us with interconnections and interdependencies among the field and between interdisciplinary fields.

Methodology

In this study, the documents were retrieved from the collection of Scopus database. This search was performed on 15th March 2024. The search query was "heart disease" OR "cardiovascular disease" AND "logistic regression" AND "prediction". A total of 2331 publications were compiled using the above query. The data was limited to articles and conference papers. Publications of all languages were considered. The resulting "CSV" file was fed into the VOSviewer (Centre for Science and Technology Studies (CWTS), Leiden University, the Netherlands) and Biblioshiny (Aria and Cuccurullo, 2017). The VOSviewer software version 1.6.19 was used to perform the bibliometric analysis in this study. The overview of the study and findings are shown in Table 1.

**Table 1:** Essential aspects of the investigation on heart disease classification using the logistic regression model

Description	Results
Search Query	( TITLE-ABS-KEY ( "heart disease" ) OR TITLE-ABS-KEY ( "cardio vascular disease" ) AND TITLE-ABS-KEY ( "logistic regression" ) AND TITLE-ABS-KEY ( prediction ) ) AND ( LIMIT-TO ( PUBYEAR , 2023 ) OR LIMIT-TO ( PUBYEAR , 2022 ) OR LIMIT-TO ( PUBYEAR , 2021 ) OR LIMIT-TO ( PUBYEAR , 2020 ) OR LIMIT-TO ( PUBYEAR , 2019 ) AND ( LIMIT-TO ( DOCTYPE , "ar" ) OR LIMIT-TO ( DOCTYPE , "cp" ) )
Timespan	2019:2023
Sources	1155
Documents	2331
Annual Growth Rate %	34.8
Document Average Age	2.45
Average Citations Per Doc	7.556
References	72205
DOCUMENT CONTENTS	
Keywords Plus (ID)	10236
Author's Keywords (DE)	4504
AUTHORS	
Authors	12150
Authors of Single-Authored Docs	28
AUTHORS COLLABORATION	
Single-Authored Docs	28
Co-Authors Per Doc	7.08
International Co-Authorships %	17.42
DOCUMENT TYPES	
Article	1881
Conference Paper	450

## Review

Results

Annual Scientific Production

**Figure 1:**
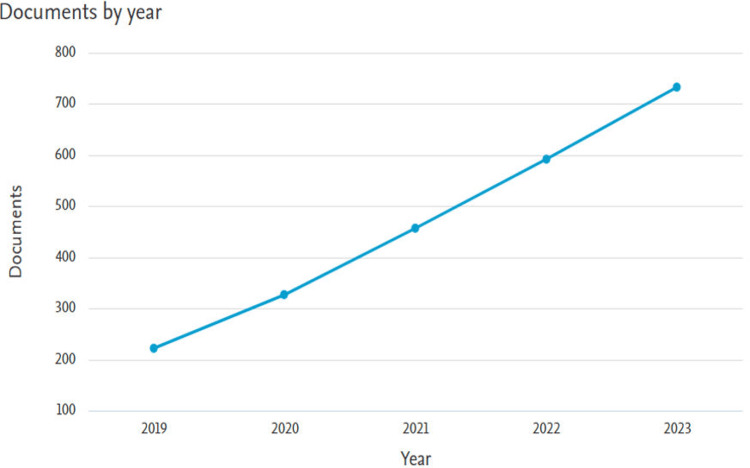
The number of documents on heart disease classification using the logistic regression model per year from 2019 to 2023.

Annual scientific production allows us to observe the growth and expansion of the field over the years. There is a steady growth in the number of papers from 2019 to 2023 from 222 in 2019 to 733 in 2023 (Figure 1). We can attribute this growth to the advancement and significant growth of ML during recent years. Also, the availability of vast amount of healthcare data from electronic healthcare records in hospitals and wearable gadgets is another reason. Additionally, the interdisciplinary nature of the field and the collaboration among data scientists, healthcare professionals, biomedical engineers, and others have also contributed to the increase in annual scientific output.

Average Citations Per Year

**Figure 2:**
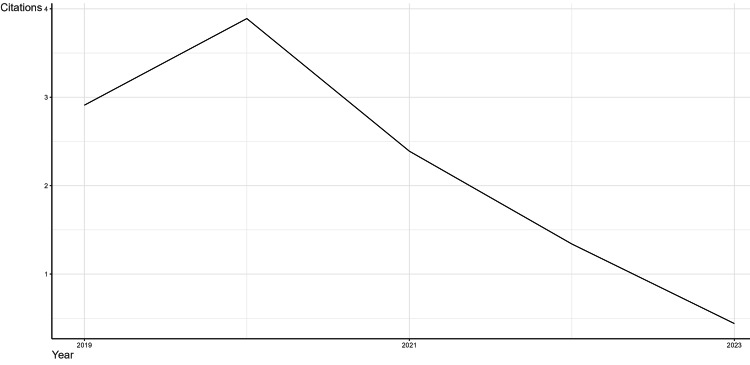
The average citations per year from 2019 to 2023 on heart disease classification using the logistic regression model represented using the tool Biblioshiny.

Average citations per year give the average times a publication has been cited in a year. This metric is very crucial for analyzing the quality of the scholarly work. It can also indicate the impact and influence of the author and affiliated institution. The annual average citations are shown in Figure 2. Although the scientific output of the field is steadily ascending, there is a decline in the number of citations from 2.91 in 2019 to 0.44 in 2023. This could indicate the field deviating from traditional methods like logistic regression with the rise of more advanced ML and deep learning techniques such as neural networks. Another reason could be the incorporation of diverse datatypes such as ECG and medical imaging data which predominantly use convolutional neural networks and recurrent networks for extraction of data. It also may be due to the fact that the field is nearing saturation; however, it should also be noted that, since the study only included publications from the last five years, citations may still require time to accumulate.

Three Field Plot

**Figure 3:**
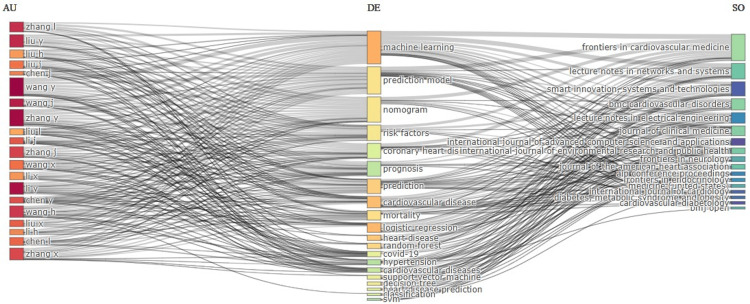
Three field plots with Author (left), Keyword (middle) and Source (right).

Three field plots are used to visualize the relationship between primary elements of three categories and how they are interconnected through a Sankey diagram. Sankey diagrams can be used to illustrate the flow of energy and materials in various networks and processes [10]. It helps to connect three metrics and depicts how they influence each other [11]. Figure 3 shows the diagram of research on heart disease classification using a logistic regression model per year from 2019 to 2023 on the relation between author (left), keyword (middle) and source (right).

The central clustering of keywords such as "machine learning," "prediction model," "nomogram," "risk factors," and "coronary heart disease" indicates that these topics are central to the research area. The left side of the plot highlights influential authors who are significantly contributing to the field, such as Zhang I, Liu Y, Liu H, Liu J, Wang Y and so on. The right side of the plot highlights key journals and sources such as *Frontiers in Cardiovascular Medicine*, *Lecture Notes in Networks and Systems*, smart innovation, systems and technologies and so on. The highlighted sources are the primary publication journals for influential research in this area. The interconnections between authors and specific keywords suggest areas of expertise and research focus for these authors. The interconnections between keywords and sources demonstrate which journals are most frequently publishing research on these topics.

Publication Output of Countries

**Figure 4:**
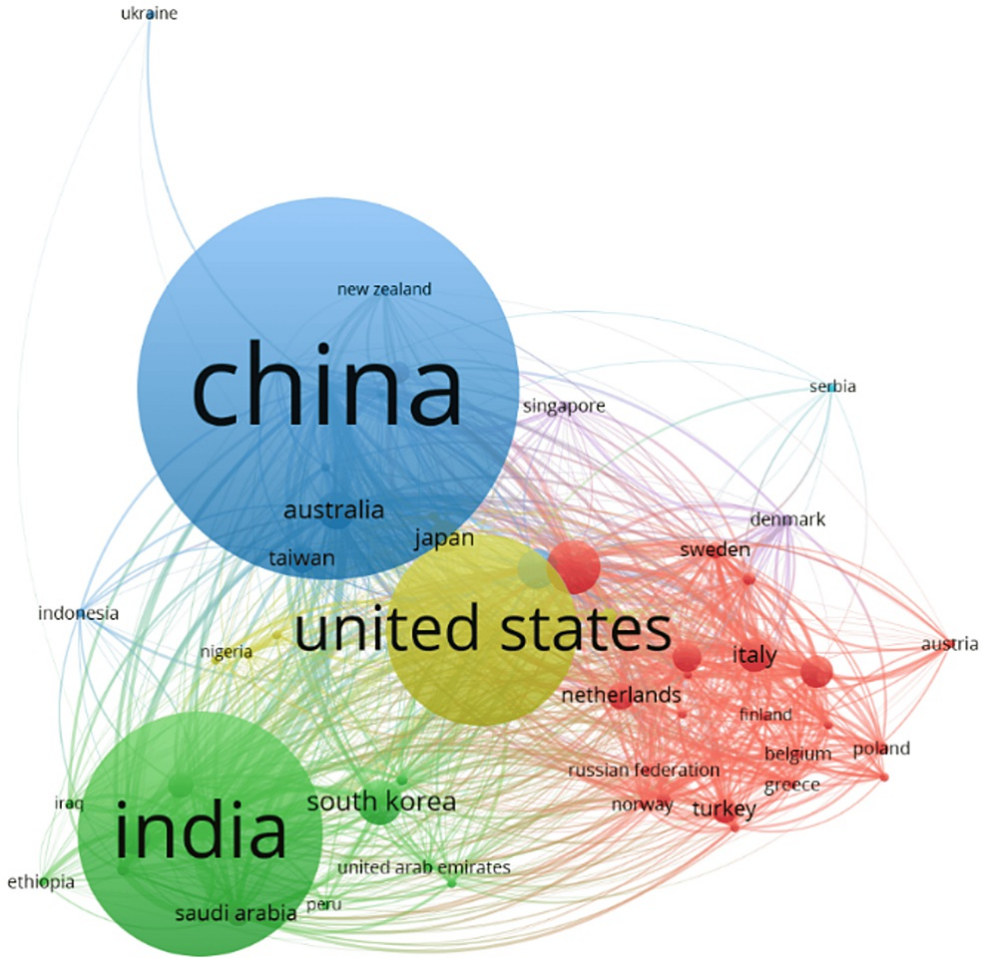
The visualization map of countries that published articles on heart disease classification using the logistic regression model.

Publication analysis of countries is a strong indicator of the amount of research work carried out by a country in a subject area [12]. By analyzing the publication patterns of countries, researchers can gain insights into the extent of research conducted in a particular field by a country [13]. This information can be used to identify the leading countries in that field and make informed decisions regarding collaborations and resource allocation. The country-wise analysis showed that China occupied the first position with 700 documents, followed by India with 447 documents. United States of America being in third position has a total of 347 documents. The country-wise visualization result is shown in Figure 4. The top five countries with the highest number of documents are given in Table 2.

**Table 2:** Top five countries ranked by the number of documents on heart disease classification using the logistic regression model.

No.	Country	No. of Documents	No. of Citations
1	China	700	5126
2	India	447	1956
3	United States	347	4098
4	United Kingdom	100	949
5	South Korea	82	1087

The domination of two countries, India and China, could be attributed to the rapid economic growth, leading to significant investment in healthcare and education sectors as well as the rise of academic institutions in those countries. The growing global influence of these countries also indicates the increased international collaborations and extensive research activities. In addition to that, the large population of the countries provides an additional advantage by having a multitude of competitive and proficient researchers.

Most Productive Authors

**Figure 5:**
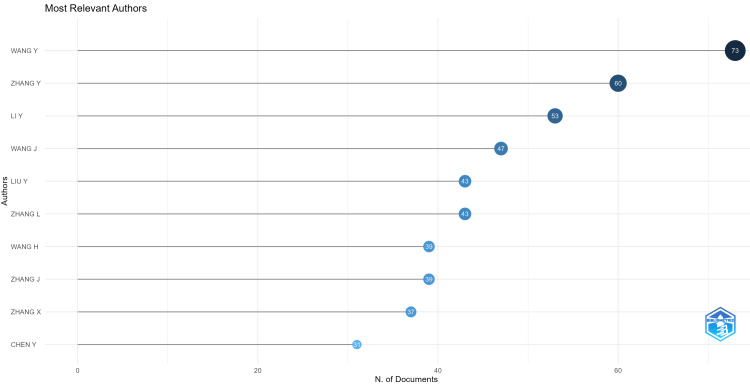
The visualization map of prominent authors who published articles on heart disease classification using the logistic regression model using the tool Biblioshiny.

We used Biblioshiny to identify the most relevant authors in the field. Wang Y topped the list with 73 documents, followed by Zhang Y with 60 documents. Li Y bagged the third position with 53 documents followed by Wang J (47) and Liu Y (43 documents) (Figure 5).

The study found that there is no overlap between the most productive authors and the authors of the most highly cited papers. This indicates that while proficient authors contribute a large volume of research, it is the works by other researchers that receive the most citations. Another reason could be the presence of the pandemic. Among the top five most cited articles, three publications are the studies related to the COVID-19 virus which were highly relevant during that time.

Most Cited Articles

**Figure 6:**
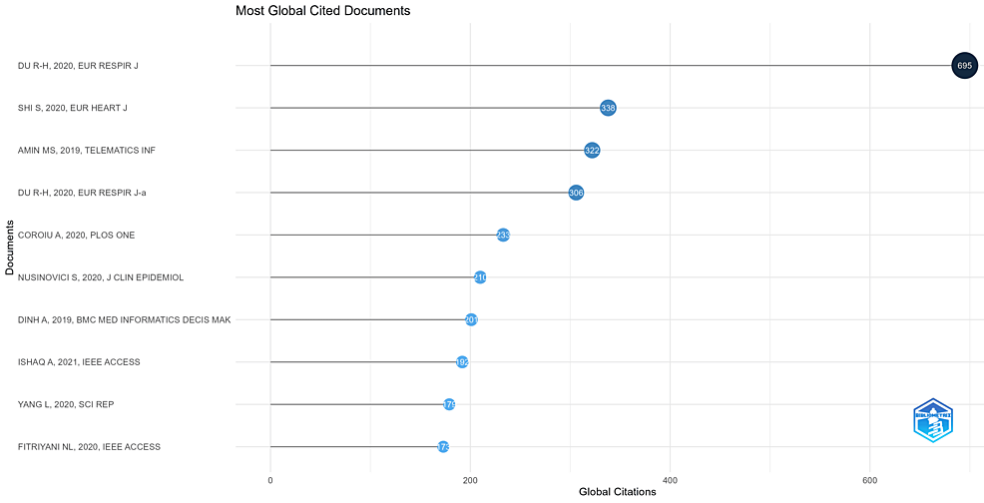
Top globally cited documents on the topic of heart disease classification using the logistic regression model.

The number of citations gives the impact of the scholarly work made in the particular field of research [14]. Out of 2331 documents under investigation, 423 documents met the requirement of a minimum of 10 citations. The top five highly cited articles are shown in Table 3. The visualization of the top globally cited documents on heart disease classification using the logistic regression model is given in Figure 6.

**Table 3:** Top five articles on heart disease classification using the logistic regression model ranked by total number of citations.

No.	Authors	Titles	Sources	No. of Citations
1	Rong-Hui Du, Li-Rong Liang, Cheng-Qing Yang, Wen Wang, Tan-Ze Cao, Ming Li, Guang-Yun Guo, Juan Du, Chun-Lan Zheng, Qi Zhu, Ming Hu, Xu-Yan Li, Peng Peng, Huan-Zhong Shi	Predictors of Mortality for Patients With COVID-19 Pneumonia Caused by SARS-CoV-2: A Prospective Cohort Study	*European Respiratory Journal*, Volume 55, Issue 5, 2020 May	695
2	Shaobo Shi, Mu Qin, Yuli Cai, Tao Liu, Bo Shen, Fan Yang, Sheng Cao, Xu Liu, Yaozu Xiang, Qinyan Zhao, He Huang, Bo Yang, Congxin Huang	Characteristics and Clinical Significance of Myocardial Injury in Patients With Severe Coronavirus Disease 2019	*European Heart Journal*, Volume 41, Issue 22, 7 June 2020, Pages 2070-2079	338
3	Mohammad Shafenoor Amin, Yin Kia Chiam, Kasturi Dewi Varathan	Identification of Significant Features and Data Mining Techniques in Predicting Heart Disease	*Telematics and Informatics,* Volume 36, March 2019, Pages 82-93	322
4	Adina Coroiu, Chelsea Moran, Tavis Campbell, Alan C. Geller	Barriers and Facilitators of Adherence to Social Distancing Recommendations During COVID-19 Among a Large International Sample of Adults	*PLOS One*, Volume 15, 2020	233
5	Simon Nusinovici, Yih Chung Tham, Marco Yu Chak Yan, Daniel Shu Wei Ting, Jialiang Li, Charumathi, Sabanayagam, Tien Yin Wong, Ching-Yu Cheng	Logistic Regression Was As Good As Machine Learning for Predicting Major Chronic Diseases	*Journal of Clinical Epidemiology*, Volume 122, June 2020, Pages 56-69	210

Contribution of Organizations

By exploring the contribution of organizations, the significance and extent of research work done by a particular organization can be identified [15]. Out of 8028 organizations, only 28 met the requirement of at least three documents. The top five organizations involved in heart disease classification using logistic regression model research are listed in Table 4. Harvard Medical School, Boston, MA, United States topped the list with papers followed by the College of Medical Informatics, Chongqing Medical University, Chongqing, China and China National Clinical Research Center for Neurological Diseases, Beijing, China with five papers each.

**Table 4:** The top organizations on the topic of heart disease classification using the logistic regression model ranked by the number of documents.

No.	Organizations	No. of Documents	No. of Citations
1	Harvard Medical School, Boston, MA, United States	6	51
2	College of Medical Informatics, Chongqing Medical University, Chongqing, China	5	9
3	China National Clinical Research Center for Neurological Diseases, Beijing, China	5	7
4	University of Colorado School of Medicine, Aurora, CO, United States	4	29
5	Medical Data Science Academy, Chongqing Medical University, Chongqing, China	4	9
6	Operation Management Office, Affiliated Banan Hospital of Chongqing Medical University, Chongqing, China	4	6

Institutional collaboration can significantly lead to rapid advancements in the field due to its ability to bring together experts from various disciplines, share infrastructure and resources, and enable large-scale studies, which could be more challenging for single institutions. For example, institutional collaborations can bring experts from various fields such as cardiology, data science, and biomedical engineering, which leads to the development of accurate prediction models. Furthermore, such collaborations often promote the wide dissemination of data and the development of novel techniques.

Keyword Analysis

**Figure 7:**
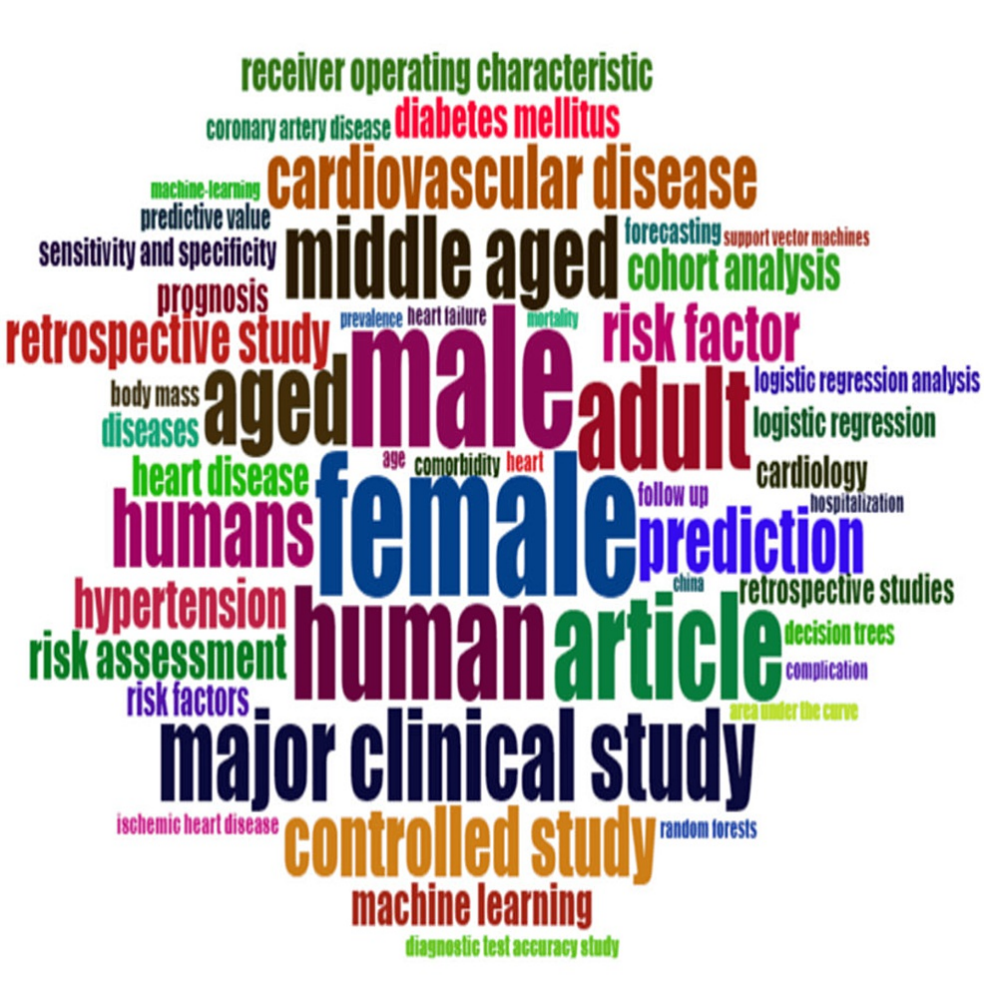
Word cloud of keywords on the topic of heart disease classification using the logistic regression model.

Keyword analysis identifies the frequency of recurring keywords in scholarly publications in the field which helps to observe the primary focus of the field and prominent and emerging themes [16]. The word cloud visualization of keywords on heart disease classification using a logistic regression model is given in Figure 7. The top five keywords by frequency count in publications are listed in Table 5.

**Table 5:** Top five keywords involved in the heart disease classification using the logistic regression model research, ranked by occurrence count.

No.	Keywords	No. of Occurrences
1	Female	2046
2	Male	1996
3	Human	1623
4	Article	1529
5	Adult	1484

The presence of frequent keywords, Female and Male, can be attributed to the focus on gender-based differences in medical research. It is crucial to address distinctions in diseases, treatments, and health results between both genders to create effective medical treatments. Also, it indicates a growing interest in gender-based research rectifying the historical bias where medical research predominantly considered male subjects only. The human keyword may indicate human-related clinical research rather than studies based on other organisms and filtering out theoretical papers. The article keyword primarily indicates the type of publication. And adult keyword indicates the age group of people under study and it aligns with the fact that heart health issues are more prevalent in the adult population.

Most Relevant Sources

**Figure 8:**
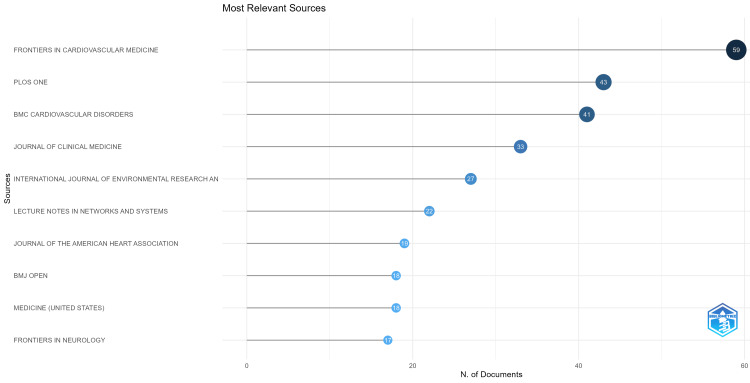
The top 10 significant sources in terms of the number of publications visualized using Biblioshiny.

There were a total of 1155 journal sources. Out of these, *Frontiers in Cardiovascular Medicine* was found to be the most significant source with 59 papers followed by *PLOS One* with 43 papers and *BMC Cardiovascular Disorders* with 41 papers. Figure 8 shows the top 10 journals that produced scholarly work on heart disease classification using the logistic regression model.

Comparing the influence and reach of these sources by impact factors, *Frontiers in Cardiovascular Medicine *holds an impact factor of 3.6, while *PLOS One* follows closely with 2.9. *BMC Cardiovascular Disorders* holds an impact factor of 2.1, and the *Journal of Clinical Medicine* leads with an impact factor of 3.9 and so on.

Most Significant Affiliations

**Figure 9:**
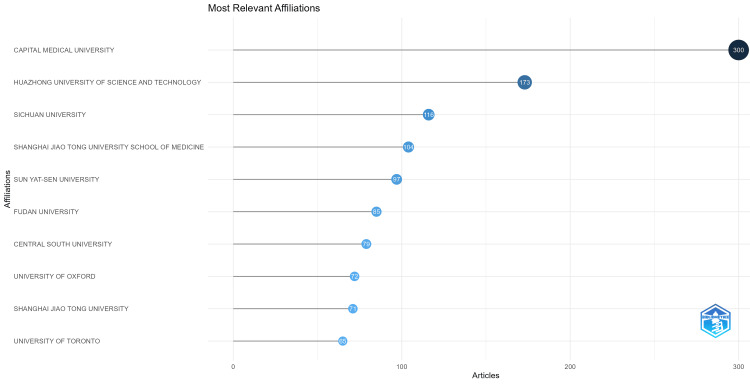
Most significant affiliations on heart disease classification using the logistic regression research visualized using Biblioshiny.

The most significant institutions involved in the research on heart disease classification using the logistic regression model are tabulated in Figure 9. Leading the list is Capital Medical University, established in 1960, with 300 papers. It is one of China's top medical academic institutions. Huazhong University of Science and Technology in China, renowned for its interdisciplinary approach combining engineering, data science, and medicine, which aids researchers in producing innovative research outputs, was found to be next on the list with 173 papers. Sichuan University is another prominent institution noted for its substantial contribution to the field, creating a strong research environment and producing 116 papers and so on. Figure 9 shows the visualization map of the most significant affiliations on heart disease classification using logistic regression research.

Analysis of Trending Topics and Thematic Maps

**Figure 10:**
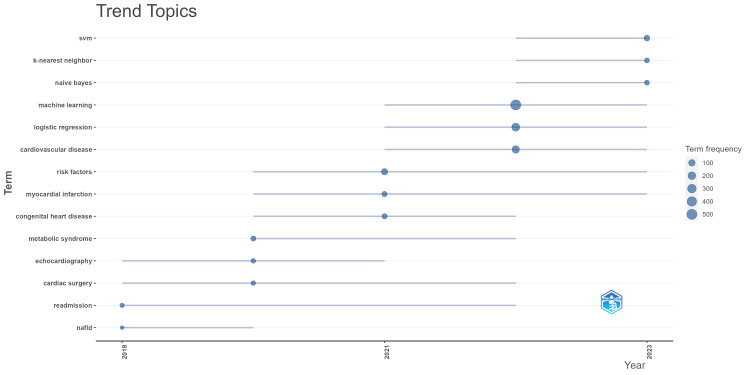
Trending topics in heart disease classification using the logistic regression research.

Analysis of trending topics based on authors' keywords from the collection is conducted, which revealed interesting patterns spanning years from 2019 to 2023. For example, in 2019, the trending topics were echocardiography, cardiac surgery, and nonalcoholic fatty liver disease (NAFLD) which are predominantly medical terms. In 2022, the most trending topics were ML, logistic regression and cardiovascular diseases and in the year 2023, it changed to support vector machines (SVM), k-nearest neighbour (KNN) and naïve Bayes which are known for its unique strengths in pattern recognition and prediction models. For example, SVM has the advantage of performing well with a larger number of features, which is useful for medical clinical research that includes numerous patient features and biomarkers. Also, SVM generalizes well to new, unseen data, which is crucial for clinical applications and so on. This shift indicates a transition into an interdisciplinary approach, highlighting the integration of advanced computational techniques into traditional medical research. By 2022, there was a definite shift towards incorporating ML into cardiovascular research. Also, the transition from general ML concepts to diving deep into specific topics indicates the development and maturation of the field. In the coming years, the transition will be towards integrating interpretability models, deep complex learning models, real-time data analysis and so on. The visualization of trending topics on heart disease classification using logistic regression research is given in Figure 10.

Thematic maps can be used to find the current themes and potential future scenarios of the field [17]. Thematic maps use keywords to find the interconnection between them to identify major themes. These themes are distinguished by their characteristics, such as density and centrality [18]. Density is shown on the vertical axis, which is the developmental degree whereas centrality is shown on the horizontal axis and is the relevance degree. These two characteristics depict the potential and significance of a specific topic. Nodes with higher frequency or connections in the thematic network have a higher impact.

**Figure 11:**
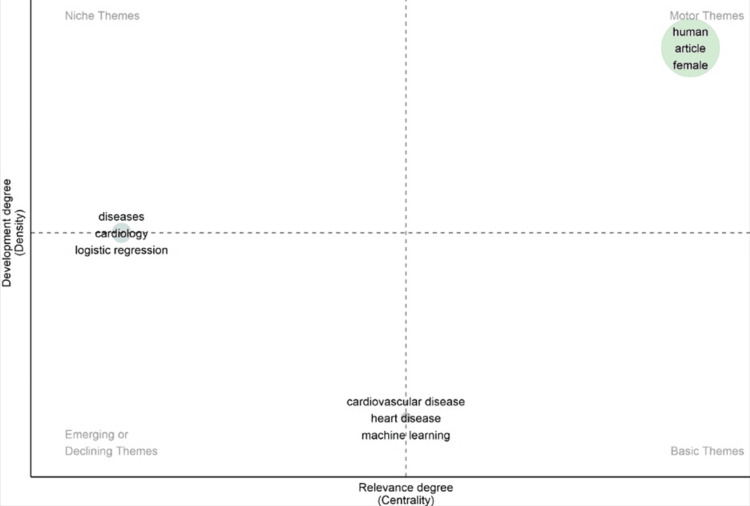
The thematic map on heart disease classification using the logistic regression research.

The map in Figure 11 is divided into four quadrants. The motor theme depicts the primary theme, niche themes depict the highly developed and specialized themes that link to the main theme, emerging or declining themes contain ascending or descending themes, and basic themes consist of foundational and cross-curricular themes.

Co-occurrence Analysis of Index Keywords

**Figure 12:**
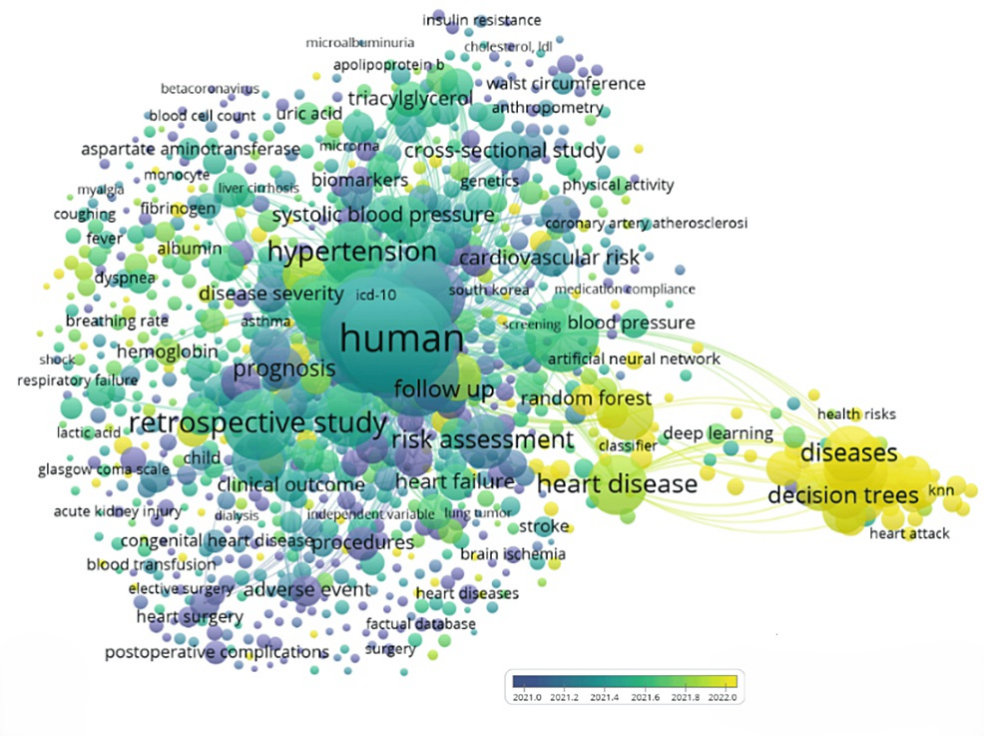
The overlay visualization map of index keyword co-occurrences on heart disease classification using the logistic regression research.

Index keywords are keywords assigned by databases or publishers to divide articles based on their content [19]. Co-occurrence analysis on index keywords analyses the frequency with which these keywords come together. The index keywords on heart disease classification using logistic regression research are selected based on five index-keyword co-occurrences. A total number of 10238 index keywords appear and 1917 meets the five co-occurrences threshold. The overlay visualization of index keywords co-occurrences is shown in Figure 12.

The interdisciplinary nature of the field is evident in the keyword co-occurrence plot. The combination of medical terms along with ML terminology hints at the efforts to improve the current heart health outcomes using advanced computational methods. Another notable fact is the presence of other disease names such as "dialysis" and "kidney injury," which highlights the interconnectedness of heart health with other organ systems, indicating that heart issues can have widespread effects on overall health. However, the smaller size of ML-related keywords suggests that, while integration with ML is emerging, it is still not the dominant focus.

Co-occurrence Analysis of All Keywords

**Figure 13:**
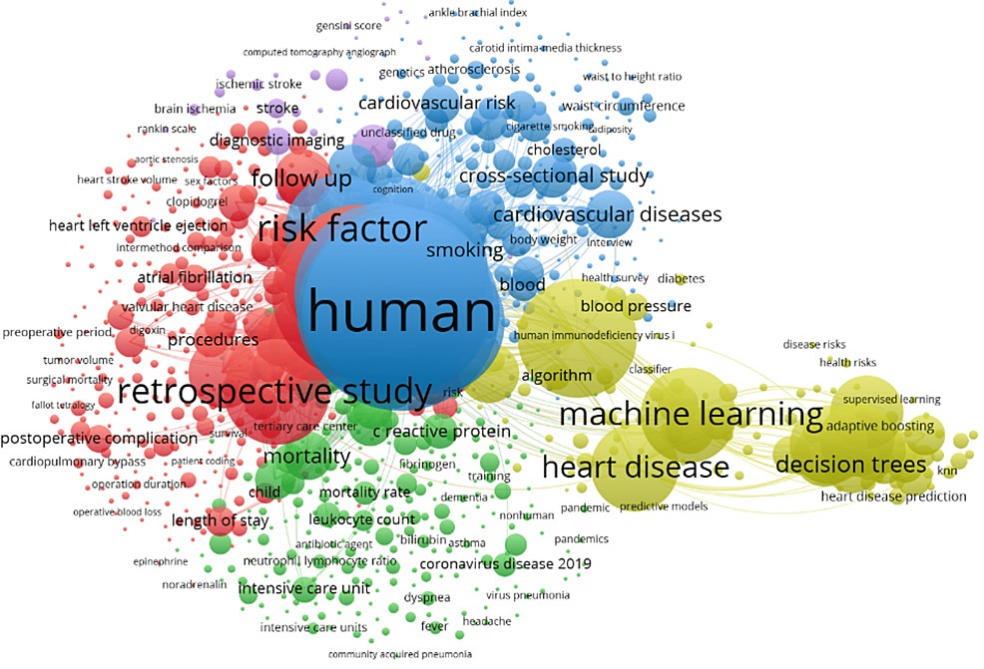
The knowledge map of the co-occurrence of keywords on heart disease classification using the logistic regression research.

The co-occurrences of all keywords in 2331 documents were also analyzed. Finding keyword co-occurrences can help you identify the patterns and relationships and interactions between different concepts in a research field. The minimum number of occurrences of a keyword in titles and abstracts was set at five in this study. The keyword analysis produced 13064 keywords out of which 2074 met the requirement of five co-occurrences. Six significant clusters were found. Cluster 1 is made up of 337 items, in which the majority of searches related to abdominal surgery, acetylsalicylic acid, acute coronary syndrome, acute heart failure, acute heart fraction, etc. The cluster 2 is made up of 258 items out of which abdominal pain, activated partial thromboplastin time, acute disease, and acute heart failure effect stood out. The knowledge map of the co-occurrence of keywords constructed is shown in Figure 13.

Gaps and future directions

The study suggests that there are significant research gaps in the fields. The size of KNN, naïve Bayes, deep learning and neural networks is small compared to other nodes. One of the primary reasons could be the well-established background and thorough validation of logistic regression in the field of medical research, leading to a substantial collection of comparative studies. In contrast, KNN and naïve Bayes encounter restrictions including computational complexity, challenges in interpretability, and assumptions regarding the independence of features, which are not well-suited for the complex characteristics of medical data. Also, deep learning and neural networks are more complex and require expertise in the field which may not be as widespread among traditional medical researchers, and they often require large amounts of labeled data, which can be a constraint in the medical field. Also, they are less transparent and the lack of interpretability is less favoured among the medical research community. Also, effective implementation of the models may require interdisciplinary collaboration, which may always not be possible. The visualization of trending topics also indicated the progression towards KNN and naïve Bayes.

The large size of traditional methods indicates the field being revolving around traditional methods. For example, we consider logistic regression, which is much trusted in the medical field due to its extensive validation in numerous studies. Additionally, the exponentiated slope coefficient offers a convenient interpretation as an odds ratio, making it easier to interpret the results in medical research. However, they have limitations in handling complex, high-dimensional data and capturing non-linear relationships. Newer techniques such as KNN, naïve Bayes, deep learning, and neural networks have the capability to overcome these limitations. These advanced methods can model complex, non-linear relationships and handle high-dimensional data more effectively. While these newer methods require rigorous validation and often involve greater complexity, their integration into clinical research could significantly improve heart disease prediction and research outputs. For example, a deep learning algorithm that incorporates MRI scans in addition to conventional risk factors has the ability to detect individuals more accurately than models that solely rely on traditional clinical information.

Healthcare professionals need to know the decision-making process of models, but the black box model structure of complex deep learning and ensemble methods makes it difficult to understand how they arrive at their predictions. Utilize interpretable models or employ post-hoc explanation tools such as SHAP (SHapley Additive exPlanations) and LIME (Local Interpretable Model-agnostic Explanations) to clarify model predictions.

The average size of body vitals shows the lack of data diversity and the field focusing on traditional risk factors such as age, gender and blood pressure levels. Traditional clinical data may not be able to identify subtle and underlying patterns. Also, they may have been derived from studies on specific populations which leads to biased predictions on different data. The diversity of the dataset is crucial for a deeper understanding of the patient profile, especially in medical research. It helps to create a more accurate and complete patient profile leading to more accurate risk assessments and predictions.

Additionally, most of the papers focus on population-level risk assessment which is static in nature. Population-level risk models are developed for general populations and may perform poorly if applied to different subgroups such as different age groups. They do not generally include lifestyle factors, ethnical varieties, etc. The necessity of personalized risk estimates is growing in order to capture the dynamic nature of disease progression. Now with the advent of wearable devices, we can monitor and assess risk factors in real time which enhances the effectiveness of preventive measures and treatments. Also, with a personalized medicine approach, where unique characteristics of each patient are considered, accuracy can be greatly enhanced.

Discussions

From 2019 to 2023, a significant growth was observed in the number of publications on heart disease classification using the logistic regression model from 222 in 2019 to 733 in 2023, demonstrating an annual growth rate of 34.8%. The Republic of China was found to be the most contributing country with 700 documents.

Considering the average number of citations per year, we can observe a peak in 2020 followed by a constant descent. The mean citations per year were 3.64 in 2018 which dropped to 2.91 in 2019. The peak in 2020 could be attributed to several factors including the huge funding and resources directed towards medical research during the COVID-19 pandemic. Also, there was increased attention toward existing health conditions, such as heart disease which also helped in attaining the peak. Furthermore, due to remote work arrangements and the slowdown or complete halt of various professional activities, researchers were able to dedicate more time to their academic and scholarly works. The descent could be due to a shift in research focus as the urgency decreased as well as the disruption caused by the pandemic. Also, the advancement in artificial intelligence and newer techniques and models may have diverted attention from traditional methods like logistic regression. The use of graphics processing units (GPUs) for training ML models has significantly reduced the time needed for model development, enabling the use of more complex algorithms and larger datasets. Also, the ascend in the number of publications could potentially lead to increased competition for citations which could be another reason for the descent.

Encouraging interdisciplinary collaboration to bridge the gap between the scientific disciplines, adding diverse data types, promoting the sharing of datasets, and open-access publishing could improve the quality of research outputs.

The most relevant author in the field is Wang Y with a total of 73 papers indicating his active engagement in the research area. Closely following is Zhang Y with 60 documents and Li Y with 43 documents. The greatest number of documents is produced by China (700), indicating the strong presence of the country in the field. The high number of citations (5126) also indicates the volume and quality of the research output. The Chinese government has significantly invested in and prioritized the healthcare sector and with policies like Healthy China 2030; the intense focus on innovation in the medical sector and research is promoted to a great extent. With advanced research infrastructure and a large pool of skilled researchers and top academic research institutions, China is equipped to lead globally in the field.

India comes up with 447 documents in the field, showing an active involvement and growing presence with a substantial number of citations (1956). The third position is bagged by USA with 347 publications yet the citation count of 4098 reveals the high quality of research work in the country.

Out of 1155 journals that published papers, 423 managed to get a minimum of 10 citations. *Frontiers in Cardiovascular Medicine* leads with 59 documents published, followed by* PLOS One* with 43, *BMC Cardiovascular Disorders* with 41, and the *Journal of Clinical Medicine* with 33. These journals, with high impact factors (ranging from 2.1 to 3.9) and fully open access status, play a crucial role in advancing heart disease classification research. Their open-access nature enables wide visibility and accessibility, helping in the rapid dissemination of scientific output and findings globally, including researchers, clinicians, and policymakers.

The most cited literature is "Predictors of Mortality for Patients With COVID-19 Pneumonia Caused by SARS-CoV-2: A Prospective Cohort Study" with 695 citations. The most productive organization is Harvard Medical School, Boston, MA, United States with six documents.

## Conclusions

Heart disease and concerned treatments are one of the prominent research areas all over the world. Using ML and artificial intelligence, the diagnosis and prediction of heart-related diseases can be enhanced considerably. Although various bibliometric studies have been conducted either on logistic regression or heart disease classification, this is the first study that conducts a comprehensive bibliometric analysis combining these two aspects. This study provides valuable insights about the field ranging from proficient institutions and authors to the anticipated trajectory of the current field in the future and geographical patterns in research productivity. It also aids in revealing the interdependencies among different areas and current trends and themes.

The results obtained will give a better understanding of the current research scenario and help future researchers explore the field of study. Furthermore, it gives clarity about the research gap in the field. Most studies focus on performance metrics, but there is a critical need to address how healthcare professionals can interpret and trust the results generated by these models. While logistic regression is generally interpretable due to its straightforward coefficients, using tools like SHAP and LIME can further enhance understanding, especially when dealing with complex models or a large number of features. Also, the need to incorporate the strength of newer technologies is significant. Exploring hybrid models that integrate logistic regression with advanced technologies such as deep learning could boost classification accuracy and performance. Ensuring the use of diverse data types will help to personalize the prediction thereby increasing the accuracy of the prediction. China is found to be the most prolific country whereas Wang Y is found to be the most relevant author. *Frontiers in Cardiovascular Medicine* is the most relevant journal and Harvard Medical School, Boston, MA, United States is the most productive organization. These findings could be of great use for healthcare professionals to implement evidence-based diagnostic models for better patient outputs. It will help policymakers to strategically fund the resources in under-researched yet important areas, and for researchers to identify major gaps and address them.
